# Manipulating microRNAs for the Treatment of Malignant Pleural Mesothelioma: Past, Present and Future

**DOI:** 10.3389/fonc.2020.00105

**Published:** 2020-02-07

**Authors:** Glen Reid, Thomas G. Johnson, Nico van Zandwijk

**Affiliations:** ^1^Department of Pathology, University of Otago, Dunedin, New Zealand; ^2^Maurice Wilkins Centre, University of Otago, Dunedin, New Zealand; ^3^The Asbestos Diseases Research Institute, Sydney, NSW, Australia; ^4^Cell Division Laboratory, The ANZAC Research Institute, Sydney, NSW, Australia; ^5^School of Medicine, The University of Sydney, Sydney, NSW, Australia; ^6^Sydney Catalyst Translational Cancer Research Centre, The University of Sydney, Sydney, NSW, Australia; ^7^Sydney Local Health District, Sydney, NSW, Australia

**Keywords:** microRNA, malignant pleural mesothelioma, tumor suppressor miRNA, oncomiR, extracellular vesicles, drug delivery, drug formulation

## Abstract

microRNAs (miRNAs) are an important class of non-coding RNA that post-transcriptionally regulate the expression of most protein-coding genes. Their aberrant expression in tumors contributes to each of the hallmarks of cancer. In malignant pleural mesothelioma (MPM), in common with other tumor types, changes in miRNA expression are characterized by a global downregulation, although elevated levels of some miRNAs are also found. While an increasing number of miRNAs exhibit altered expression in MPM, relatively few have been functionally characterized. Of a growing number with tumor suppressor activity *in vitro*, miR-16, miR-193a, and miR-215 were also shown to have tumor suppressor activity *in vivo*. In the case of miR-16, the significant inhibitory effects on tumor growth following targeted delivery of miR-16-based mimics in a xenograft model was the basis for a successful phase I clinical trial. More recently overexpressed miRNAs with oncogenic activity have been described. Many of these changes in miRNA expression are related to the characteristic loss of tumor suppressor pathways in MPM tumors. In this review we will highlight the studies providing evidence for therapeutic effects of modulating microRNA levels in MPM, and discuss these results in the context of emerging approaches to miRNA-based therapy.

## Introduction

Numerous studies in the last decade have shed light on the characteristic changes in microRNA (miRNA) expression in malignant pleural mesothelioma (MPM). MiRNAs are an important class of non-coding RNA that post-transcriptionally regulate the expression of most protein-coding genes (1). In addition to central roles in normal biology, their aberrant expression in tumors contributes to all of the hallmarks of cancer (2). In common with other tumor types, changes in miRNA expression in MPM are characterized by a global downregulation, although elevated levels of some miRNAs are also found (3). These changes have been explored in order to identify new biomarkers, as well as to better understand the role of miRNAs in MPM biology and to evaluate their potential as therapeutic targets for MPM (3, 4). In this review, we focus on miRNAs for which biological activity in MPM has been demonstrated, in particular highlighting *in vivo* findings and clinical studies. These will be discussed in relation to the development of miRNAs (and siRNAs) as therapies for cancer and other diseases.

## Modulating microRNA Levels in MPM

### Tumor Suppressor miRNAs—Early Studies

Multiple miRNAs are downregulated in MPM samples when compared with non-neoplastic control tissue (see reviews) but relatively few have been characterized functionally (Table 1). Initial studies reported modest *in vitro* tumor suppressor activity of miR-29c-5p, miR-31-5p, and miR-145-5p, among others. In a series of surgical samples, lower levels of miR-29c-5p (the rarer passenger strand of miR-29c) were associated with poor prognosis (16). Using a mimic to restore expression levels revealed miR-29c-5p to have modest tumor suppressor activity in two MPM cell lines *in vitro*, by inhibition of proliferation and migration/invasion. The same mimic led to downregulation of the DNA methyltransferases DNMT3A and DNMT3B, as well as increasing expression of upstream signaling molecules including adiponectin. In a subsequent study, the same group demonstrated frequent loss of miR-31 expression in MPM cell lines due to co-deletion of *MIR31HG* with the *CDKN2A* locus (17). Re-expressing miR-31 with a mimic again led to modest inhibition of proliferation, clonogenic growth and migration/invasion in the same two MPM cell lines. Loss of miR-31 further correlated with the elevated expression of cell cycle and replication-associated genes.

**Table 1:** Dysregulated miRNAs with biological activity in MPM.

	**Expression change in MPM**			**Activity**				
**microRNA**	**Cells**	**Tumors**	**Prognostic value?**	***In vitro***	***In vivo***	**Experimentally validated function(s)**	**TS or oncomiR?**	**References**
Let-7a	N.D.	N.D.	N.D.	√	–^¶^	Induced by EphrinA1; inhibits RAS	TS	(5)
miR-1-3p	N.D.	↓	N.D.	√	–	Inhibits proliferation and migration/invasion; targets PIM1	TS?	(6, 7)
miR-15a-5p	↓	↓	None	√	–	Inhibits growth of MPM cells	TS	(8)
miR-15b-5p	↓	↓	None	√	–	Inhibits growth of MPM cells	TS	(8, 9)
miR-16-5p	↓	↓	None	√	√^*^	Tumor suppressor functions; downregulates CCND1, BCL2, and PD-L1	TS	(8, 9)
miR-17-5p	↓	↓	High Exp = SS	√	–	Inhibits migration; targets KCNMA1	TS	(10)
miR-18a-5p	↑	N.D.	High Exp = SS	√	–	Antimir causes modest growth inhibition; targets PIAS3	OncomiR	(11)
miR-21-5p	N.D.	↑	High Exp = SS	√	–	Mimic causes modest growth inhibition; targets mesothelin	TS?	(12–14)
miR-24-3p	↑	↑	N.D.	√	(√)	Promotes migration and tumor growth in mice; targets CGN	Oncomir	(15)
miR-29c-5p	↓	N.D.	High Exp = LS	√	–	Mimic inhibits growth and migration; targets DNMT1/3A	TS	(16)
miR-31-5p	↓	↓	High Exp = SS	√	–	Mimic inhibits growth and migration; targets PPP6C; role in drug resistance	TS?	(17–19)
miR-34a-5p	↓	↓	N.D.	√	√^i^	Lost in genetically modified mouse model; targets c-Met	TS	(20–23)
miR-34b-3p	↓	↓	N.D.	√	√	Inhibits MPM growth, enhance radiosensitivity; inhibitors promote mesothelial proliferation	TS	(21, 22, 24–26)
miR-34c-5p	↓	↓	N.D.	√	√	Inhibits MPM growth, enhance radiosensitivity; inhibitors promote mesothelial proliferation	TS	(21, 22, 24–26)
miR-126-3p	↓	↓	N.D.	√	(√)	Induced by oxidative stress; alters metabolism, inhibits respiration, angiogenesis; targets IRS1	TS?	(27, 28)
miR-137-3p	↑/↓	↑/↓	High Exp = SS	√	–	Inhibits growth and migration/invasion; targets YB-1	TS	(29)
miR-145-5p	↓	↓	N.D.	√	(√)	Inhibits clonogenicity and migration, sensitizes to pemetrexed; regulates OCT4	TS	(30)
miR-182-5p	↑	N.D.	N.D.	√	–	Overexpressed, antimir inhibits growth; targets FOXO1	OncomiR	(6, 31)
miR-183-5p	↑	N.D.	N.D.	√	–	Overexpressed, antimir inhibits growth; targets FOXO1	OncomiR	(6, 31)
miR-193a-3p	↓	↓	High Exp = LS	√	√^*^	Tumor suppressor; targets MCL-1 and PD-L1	TS	(9, 32)
miR-193a-5p	↓	↓	High Exp = LS	√	–	Tumor suppressor function	TS	(32)
miR-205-5p	↓	E>non-E	N.D.	√	–	Involved in EMT, affects migration; targets ZEB1 and ZEB2	TS	(33)
miR-206-3p	N.D.	↓	High Exp = LS	√	√	Inhibits growth and migration/invasion; targets KRAS/CDK4/CCND1	TS	(7, 34)
miR-223-3p	↓	↓	N.D.	√	–	Inhibits migration; targets STMN1	TS	(35)
miR-215-5p	↓	↓	High Exp = LS	√	√^*^	P53 regulated, mimic inhibits growth; targets MDM2	TS	(36)
miR-302b-3p	N.D.	N.D.	N.D.	√	–	Induced by EphrinA1, inhibits proliferation; targets MCL1	TS	(37)

√, activity shown experimentally; –^¶^, no experimental evidence of activity; √^*^, in vivo activity following systemic administration; (√), in vivo activity consists of tumour cells transfected pre-implantation; √^i^, in vivo activity inferred by loss of function; ↑, increased expression; ↓, decreased expression; ↑/↓, expression either up- or downregulated; SS, short survival; LS, long survival; E, epithelioid MPM; non-E, non-epithelioid MPM.

Following these initial studies, *in vitro* tumor suppressor activity in MPM has been ascribed to a growing number of miRNAs (Table 1). A well-characterized example is miR-145. Restoring expression of miR-145, one of a number of miRNAs found to be down-regulated in a small series of MPM tumor samples, inhibited proliferation and migration, and induced senescence (30). MPM cells transfected with a miR-145 mimic before implantation into SCID mice formed fewer and smaller tumors compared with control mimic-transfected cells. At least part of the activity of miR-145 was linked to its targeting of OCT4, a gene involved in the hypermigratory phenotype of aggressive tumors via control of the epithelial-to-mesenchymal transition (EMT). Another miRNA influencing EMT in MPM is miR-205. In a comparison of epithelioid and non-epithelioid tumors, EMT regulators ZEB1 and ZEB2 were expressed at lower levels in biphasic and sarcomatoid tumors, along with a decrease in epithelial markers (33). These changes corresponded with a decrease in miR-205 in MPM tumor samples and cells lines. Transfecting MSTO-211H cells with a miR-205 mimic reduced ZEB1/2 expression and inhibited migration and invasion.

### Tumor Suppressor miRNAs—*in vivo* Activity

Despite the increasing number of miRNAs exhibiting tumor suppressor function in MPM, only a handful have been demonstrated to have *in vivo* activity in clinically relevant models. In the case of miR-16-5p and miR-193a-3p, the growth inhibitory activity of both *in vitro* was confirmed in xenograft tumor models in two independent studies (8, 32). In these studies, mimics were loaded into bacterial minicells and targeted to MSTO-211H-derived xenografts via an EGFR-specific antibody. The minicells (known as EDVs) are formed through the asymmetric cell division of bacterial, and were previously used to deliver drugs and siRNAs to tumor xenografts (38, 39). Minicell delivery is achieved through a combination of passive accumulation via the leaky vasculature of the tumor and specific targeting using antibodies to a cell-surface antigen (EGFR) in the tumor. In both studies, systemic administration of mimic-loaded minicells led to significant inhibitory effects on tumor growth (8, 32). This was likely to be at least in part due to the inhibition of anti-apoptotic and cell cycle genes demonstrated *in vitro* in these studies.

Results from these studies laid the foundation for the phase I MesomiR-1 trial, investigating the safety and optimal dose of a miR-16-based mimic delivered in anti-EGFR antibody-targeted bacterial minicells, dubbed TargomiRs. The mimic was a novel sequence based on the consensus sequence of the miR-15 family (all of which are downregulated in MPM), which was shown to inhibit tumor xenograft growth at a similar level to native miR-16-5p (40). This trial of 27 patients demonstrated safety of the treatment as well as initial signs of activity, with one objective response (41) and stable disease in a further 15 patients (42). With miR-16-5p also impacting response to chemotherapy (8) and contributing to PD-L1 regulation (9) *in vitro*, restoration of miR-16-5p levels in combination with chemo or immunotherapy are potential future applications of this approach. In addition, recent demonstration of effective delivery of doxorubicin to MPM xenograft tumors using a mesothelin-specific antibody (43) further expands the scope of possible future trials.

Other miRNAs shown to exhibit pronounced tumor suppressor activity, including miR-137-3p and miR-193a-3p, are further candidates for clinical development using minicells. In the case of miR-193a-3p, minicell-mediated delivery inhibited tumor growth to a similar extent as miR-16-5p (32). In addition, both the 5p and 3p arms of miR-193a have growth inhibitory effects in MPM (32) and other cancers (44, 45), meaning that delivery of a mimic with two active arms would potentially increase the activity. The lower levels of both arms of miR-193a recently found to be associated with shorter overall survival in the TCGA study (46) (see below) lend support to this notion. A miR-137-3p mimic also led to pronounced inhibition of proliferation and migration in the majority of MPM cell lines tested (29). These phenotypes appeared to be predominantly due to miR-137-3p-mediated suppression of *YBX1*, previously identified as an oncogene in a range of cancer types, as there was no evidence of additivity when miR-137-3p was used in combination with a YBX1-specific siRNA (29).

While minicells remain the most clinically advanced approach to mediate systemic delivery of miRNA mimics, other vehicles have been regularly employed in preclinical cancer studies to deliver miRNAs and siRNAs (47). At this stage, however, we are not aware of any that have been tested in MPM. An early publication demonstrating the tumor suppressor activity of a miR-34b/c construct (24) was followed up by a short report describing *in vivo* delivery of an adenoviral vector expressing miR-34b/c (25). In this study, intratumoral injection of the adenoviral construct led to increased miR-34b/c expression in xenograft tumors and significant growth inhibition. More recently, atelocollagen was used to successfully deliver a miR-215-5p mimic in xenograft models of MPM (36). This study, based on the hypothesis that the well-known retention of functional p53 in MPM tumors that was recently confirmed by NGS studies (46, 48), could represent a molecular vulnerability. The expression of the p53-regulated miRNAs of the miR-192/194/215 family were assessed in MPM samples and high levels of miR-215-5p were found to be associated with increased overall survival. Mimics of all three family members were associated with growth inhibition, with miR-215-5p more effective than miR-194 or miR-192, the latter consistent with previous observations (32). The inhibitory effects of miR-215-5p were associated with decreased MDM2 protein levels and consequently an increase in p53 and its downstream effectors including p21, Bax and Puma (36). Moreover, the miR-215-5p mimic-mediated activation of p53 also caused an increase in miR-145-5p, the tumor suppressor miRNA discussed in the previous section (30). These *in vitro* studies were expanded to test miR-215-5p *in vivo* using a mimic complexed with atelocollagen to mediate local delivery. Peritumoral injection of this complex in a subcutaneous xenograft model reduced tumor volume, induced apoptosis and—importantly—increased levels of miR-215-5p in the tumor. Intrapleural administration reduced growth of orthotopic xenografts and improved the survival of tumor-bearing mice. This latter result is very relevant to MPM, where intrapleural drug delivery has been used in experimental treatment.

### Oncogenic miRNAs

A number of miRNAs are consistently found to be upregulated in certain cancer types, where they have cancer promoting function and have been termed oncomiRs. In contrast to the use of mimics to restore levels of tumor suppressor miRNAs downregulated in MPM, inhibition of overexpressed oncogenic miRNAs with antisense oligonucleotides is an alternative strategy for modulating miRNA levels. This approach is attractive as it may be amenable to local delivery, avoiding the problems associated with tumor targeting via systemic administration. While the number of miRNAs found to be consistently overexpressed in MPM is relatively small, recent studies suggest that their inhibition can have profound effects on MPM growth. One such example was the report of the effects of inhibiting the overexpressed miR-182-5p and miR-183-5p (31). They are upregulated in MPM cell lines where they promote proliferation and invasion, at least in part due to suppression of FOXO1. Reducing their levels with miRNA inhibitors reversed these effects, with dual inhibition showing additive effects. An oncogenic role for miR-182-5p was first demonstrated in melanoma, in which this miRNA enhances migration, invasion and metastasis via inhibition of FOXO3 and MITF (49). Upregulation of miR-182 in melanoma is due to amplification (at 7q31) of a miRNA cluster which also contains the related miR-183 and miR-96. As this region appears to be more frequently lost in MPM, the mechanism for overexpression remains to be determined.

Another miRNA with oncogenic activity in MPM is miR-24-3p, which was identified via a screen of polysome-associated miRNAs and is upregulated in cell lines and tumor samples (15). This miRNA regulates a range of genes involved in cell adhesion and communication, many of which are associated with good prognosis, and miR-24-3p knockdown reduced migration and invasion *in vitro* and *in vivo*. Although the targets of miR-24-3p identified in this study had no obvious link to MPM biology, it is intriguing that in other cancers miR-24-3p regulates both transcripts produced by the *CDKN2A* locus. Moreover, miR-24 is part of the miR-23a/24-2/27a cluster that is regulated by c-Myc and contributes to metastasis in breast cancer (50), and miR-23a and miR-27a upregulation was previously linked to loss of expression of the tumor suppressor ZIC1 (51). Whether other well-known oncogenic miRNAs such as miR-155, and miR-10b promote MPM tumor progression remains to be seen, but the initial results with miR-182-5p, miR-183-5p and miR-24-3p warrant further pre-clinical development.

### miRNAs With Unexpected Activity in MPM

**Figure 1:**
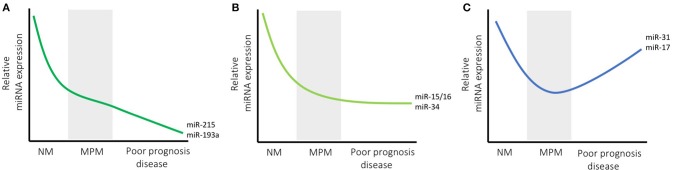
microRNA expression changes with disease course in MPM. The expression of most miRNAs is lower in MPM than normal mesothelium (NM) levels, and is shown schematically for three representative groups (levels are shown relative to NM, and are in arbitrary units for illustrative purposes). Some miRNAs are found at lower levels in tumors with poor prognosis (e.g., miR-215 and miR-193a) which may indicate a continuing gradual decrease in expression with tumor progression (indicated by decreasing levels in **A**). Others, such as miR-15/16 and the miR-34 family are consistently decreased in MPM samples but do not appear to have prognostic value, suggesting they do not change with advanced stage **(B)**. Another group, exemplified by miR-31 and miR-17, exhibit lower levels in MPM compared with NM, but are also higher in patients with shorter survival, possibly indicating an increase in expression with tumor progression **(C)**.

Recent studies suggest that miRNAs with oncogenic function in other tumor types may have variable function in MPM. In addition, several miRNAs that are reported to be downregulated in MPM compared with control tissue are nonetheless associated with poor prognosis in tumors with higher than median expression (Figure 1). The case of miR-21 is a prominent example of an oncogenic miRNA with unexpected function in MPM. This miRNA is upregulated in numerous tumor types where it is associated with multiple oncogenic functions (52). In MPM, high expression of miR-21-5p in tumor samples was associated with poor prognosis in a series of surgical samples (12). MiR-21-5p was also detected in MPM but not normal tissue by *in situ* hybridization, and was inversely correlated with expression of its target gene PDCD4 (13). In light of these observations, it is surprising that the only study to date to assess miR-21-5p activity in MPM suggests that it has modest tumor suppressor function. In a study designed to identify regulators of the MPM marker mesothelin (MSLN), both miR-21-5p and miR-100-5p were found to interact with the *MSLN* 3'UTR (14). Further experimentation revealed that a miR-21-5p mimic led to modest but significant inhibition of proliferation in two MPM cell lines, with a more pronounced reduction in colony forming ability. The authors ascribed this observation to a tumor suppressor effect that was previously observed following MSLN silencing (53).

Studies from two independent laboratories also suggest that members of the miR-17~92 polycistron, generally considered to be oncogenic (54), appear to have inconsistent functions in MPM. The first used bioinformatics to look for enriched microRNA binding sites in genes exhibiting upregulated mRNA expression, and found that miR-17 and miR-30 were both overrepresented (10). The upregulated mRNA expression was correlated with downregulation of members of the miR-17 and miR-30 seed families in both MPM cell lines and tumor samples compared with controls, and a miR-17-5p mimic reduced MPM cell migration corresponding to the downregulation of the KCa1.1 potassium channel. This result was somewhat unexpected as high levels of miR-17-5p and miR-19b-3p were associated with shorter survival in MPM patients undergoing surgery (12). In contrast, a third miRNA from the miR-17~92 cluster, miR-18a, was found to have modest oncogenic activity in MPM (11). In this study, analysis of RNA-seq data from the TCGA study revealed that high expression of miR-18a, but not others from this cluster, was associated with shorter survival. Antisense inhibition of miR-18a led to a small but significant decrease in the viability of MPM cells. The apparent discrepancy of these results may be due to the complex processing of the complex sequential processing of the 6 mature miRNAs in the polycistron, which are known to be expressed at variable levels in cells (55). Together, these results provide evidence for MPM-specific activity of mature miRNAs from the miR-17~92 cluster and warrant further investigation.

A further example of apparent inconsistencies between miRNA expression levels and activity in MPM was found in the case of miR-137-3p. Expression of this miRNA was found to be highly variable in normal mesothelium and to a greater extent in tumor samples, where evidence for both very high and very low expression was observed (29). This contrasts with most studies of miR-137-3p in cancer, where it is almost always downregulated (56). A similar range of expression was found in MPM in cell lines compared with the mesothelial control MeT-5A, but whether this correlates with the expansion of a variable nucleotide tandem repeat (VNTR) upstream of miR-137 implicated in altered processing (57) was not tested. In the series of 115 patients analyzed, high expression (defined as >2-fold increase compared with median) of miR-137-3p was associated with shorter survival. Surprisingly, an antisense inhibitor of miR-137-3p had no effect on growth whereas a mimic significantly inhibited proliferation and migration/invasion in most MPM cell lines, including those with high endogenous expression.

Another miRNA with apparent discrepancies between expression levels and functional activity is miR-31-5p. Previous studies have revealed tumor-specific functions of this miRNA, with both tumor-suppressor and oncogenic properties being observed (58). As described in the previous section, loss of miR-31-5p expression was originally linked to its tumor-suppressor activity in MPM (17). Intriguingly, this miRNA was more highly expressed in sarcomatoid tumors, albeit in a small sample set (18), and also contributed to cisplatin resistance in MPM cell lines *in vitro* (19). Whether this miRNA is solely tumor suppressive in MPM, or its activity changes over the course of the disease or in different histological subtypes, is still an open question. In contrast to downregulation in MPM, miR-31-5p was shown to be overexpressed in both mouse and human lung cancers (59), and to exhibit oncogenic activity in lung cancer cell lines (59) and in xenografts (60), with the latter observation linked to its control of BAP1 expression (60). Furthermore, a miR-31-5p antimiR repressed esophageal tumor growth *in vivo* (61), whereas in breast cancer, miR-31-5p contributed to the maintenance of the stem cell compartment and miR-31 KO compromised breast cancer tumorigenesis (62).

### Pathways Commonly Dysregulated in MPM Alter miRNA Levels

**Figure 2:**
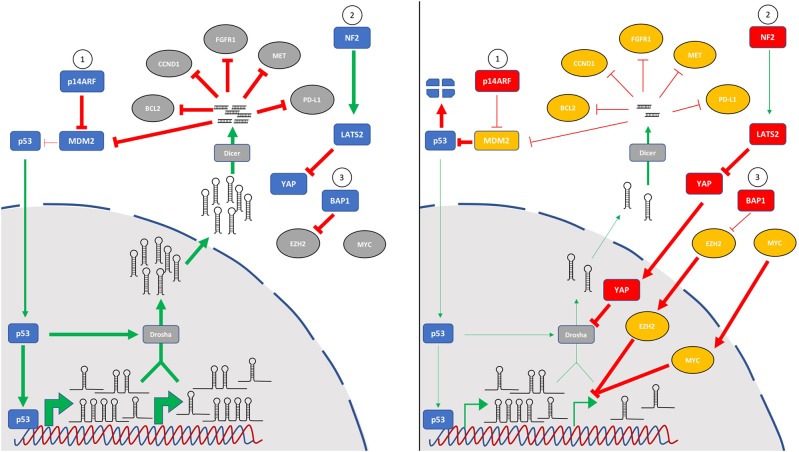
The key pathways most frequently mutated in MPM all contribute to global downregulation of microRNA levels. Tumor suppressor miRNAs are often downregulated in MPM, and several mechanisms appear to play a role in this observation. In addition to specific control of microRNA transcription, other pathways are involved in indirect control of microRNA levels via effects on processing. In mesothelial cells (left panel), the p53 pathway (1) is intact, and cell stress induces microRNA levels via direct transcription as well as by p53-induced pri-miR processing. Similarly, normal signaling of the Hippo pathway (2) through NF2 and LATS2 phosphorylates YAP1 which is retained in the cytoplasm. BAP1 deubiquitinase activity (3) destabilizes EZH2, a key component of the polycomb repressor complex 2. In mesothelioma (right panel), frequent mutation or deletion of the *CDKN2A* locus leads to loss of p14ARF and increased MDM2-mediated p53 degradation. Loss of function mutations of NF2/Merlin or LATS2 (or gain-of-function mutations of YAP) dysregulate Hippo signaling leading to accumulation of YAP in the nucleus where it can inhibit the microprocessor complex via interaction with Drosha. Inactivating BAP1 mutations prevent control of EZH2 which can alter miRNA levels. Additionally, MYC amplification or mutation can lead to transcriptional suppression of multiple miRNA genes. Whether these changes are also involved in the upregulation of oncogenic miRNAs in MPM remains to be determined.

As the number of miRNAs known to be altered in MPM continues to grow, it is interesting to note that critically dysregulated pathways in this disease converge on miRNA biology (Figure 2). Recent reports in MPM combined with earlier studies investigating the mechanistic basis of global downregulation of miRNA expression implicate the p53 tumor suppressor response as a key effector influencing miRNA levels. While MPM is unusual among solid tumors in that it generally retains wild-type p53, the p53 response is compromised by frequent loss of the upstream regulator p14ARF (via *CDKN2A* deletion) leading to upregulated MDM2 levels and increased p53 degradation. This was exploited in the study of local delivery of miR-215-5p mimic discussed earlier (36), as miR-215 is both a direct transcriptional target of p53 and a regulator of MDM2, thus forming a positive feedback loop. The study further showed a miR-215-5p-mediated upregulation of miR-145, another target of p53. Although not evaluated, it is likely that this treatment would also result in increased expression of other miRNA targets transcriptionally regulated by p53 such as miR-34a. This would be consistent with results from a mouse model of a partial *Cdkn2a* knockout, in which miR-34a suppression contributed to elevated c-Met (20). As well as direct targets, p53 is also implicated in the global downregulation of miRNAs in two important ways. First, p53 interacts with the Drosha processing complex to stimulate conversion of pri-miRNA transcripts into pre-miRs in colorectal cancer cells, thereby enhancing the maturation (without affecting transcription) of multiple tumor suppressor miRNAs, including miR-16, miR-15a and miR-145 (63). Second, because miR-145 targets c-Myc, loss of p53-regulated miR-145 expression has the added effect of relaxing post-transcriptional control of c-Myc (64). This in turn results in the transcriptional suppression of multiple miRNAs by c-Myc, including miR-15a, miR-16, miR-34a and the miR-29 family (65). This relationship was recently demonstrated directly for miR-16 in MPM (21).

Added to the central role played by dysregulated p53 activity, Hippo signaling is also implicated in altering miRNA levels in MPM. This pathway is frequently compromised in MPM through a combination of NF2 and LATS2 mutation and YAP activation (66). Like p53, this pathway stimulates maturation of primary miRNA transcripts via interaction with the Drosha processing complex (67). At low cell density, suppressed Hippo signaling culminates in nuclear localization of YAP and cellular proliferation, which is in part due to an interaction with the microprocessor protein p72 which reduces pri-miRNA processing (67). At high cell density, YAP is sequestered in the cytoplasm, the microprocessor is active and miRNA levels increase markedly. RNAi-mediated silencing of NF2 or LATS2 led to similar decreases in miRNA expression as knockdown of Drosha or p72, and affected miRNAs included let-7, miR-34a and miR-15a (67)—all found at reduced levels in MPM. Moreover, let-7 and miR-34a also target c-Myc, further exacerbating miRNA disequilibrium (68, 69). The more recently identified BAP1 mutations common in MPM (70, 71) also have a component of miRNA dysregulation. Loss of BAP1 function in MPM leads to increased expression of the polycomb repressor complex component EZH2 (72). This is consistent with the previous observations that frequent overexpression of EZH2 in MPM correlates with a decrease in levels of miR-26a and miR-101 (73), and miR-26a directly targets EZH2 in a range of cancer types (74). In turn, miR-26a is a direct target of c-Myc (75) and is downregulated in multiple cancer types. Moreover, miR-31 loss – as discussed earlier, a common event in MPM—leads to EZH2 upregulation in melanoma (76). Taken together, mutations in these three signature pathways are likely to be significant contributors to the global miRNA downregulation found in MPM.

## Future Prospects

After a decade of research into the role of miRNAs in the biology of MPM, their potential value as biomarkers and therapeutic targets is no longer in question. Initial clinical experience from the MesomiR-1 trial suggests that miRNA modulation is safe and has the potential to alter the course of disease. With the FDA approval in August 2018 of patisiran, the first ever siRNA-based drug, gene silencing has finally reached the clinic. At the time of writing at least 20 siRNAs are being evaluated in clinical trials (47, 77). However, a number of questions remain to be answered. For instance, while many miRNAs show biological activity in MPM models, other (better) targets with more pronounced tumor suppressor function in MPM may exist. More importantly, the effective delivery of nucleic acid-based drugs in general, and miRNA mimics in particular, is a problem that is far from solved. Below we discuss these two outstanding questions and how their answers may contribute to the development of new therapies for MPM.

### Additional Targets From Genomic Studies

The recent analysis of the 74 MPM samples completed by the TCGA was the first to comprehensively analyse miRNA expression in a large series of tumor samples using RNA-seq (46). Unsupervised clustering of these data revealed 5 subtypes that were associated with 5-year survival. The subgroup with the longest survival had significantly higher expression of a number of miRNAs previously identified to have tumor suppressor activity in MPM, including both miR-193a-5p and miR-193a-3p arms of miR-193a (discussed above), as well as several miR-29 family members. The prognostic value of the miR-29 family is consistent with the earlier study by Pass et al. who linked miR-29c-5p to longer survival. Their miR-29c-5p mimic inhibited growth and downregulated the DNA methyltransferases DNMT3A and DNMT3B. Overexpression of these genes in lung cancer was previously linked to reduced expression of the miR-29 family in lung cancer (78), however this was due to highly conserved targeting by the 3p arms rather than the rarer 5p arm. Similarly, the apoptosis-related gene *MCL1* and collagen genes involved in metastasis are also targeted by this family in cancer (79). As both studies used early versions of microRNA mimics it is possible that one or both were based on a pre-miR mimic containing both 5p and 3p arms. The miR-29 family also indirectly increases p53 activity by suppressing p85 and CDC42, negative regulators of p53 (80). These observations suggest that revisiting the role of the miR-29 family in MPM could reveal broader activities. Moreover, the TCGA analysis revealed a number of miRNAs with prognostic value but no known functional role in MPM such as miR-100-5p and miR-148b-3p. As these have well-characterized tumor suppressor activity in other tumor types (81, 82), they represent additional candidates for follow-up studies. In addition, histological subtype is an important determinant of MPM biology and the different subtypes are likely to be characterized by differences in miRNA expression, as mentioned in previous sections. Confirmation of the role played by miRNAs in the aggressive nature of sarcomatoid tumors awaits comparative analysis of a larger number of samples of this type.

### Mesothelioma-Specific miRNA Expression

For a miRNA to make an effective biomarker or therapeutic target in MPM, it would ideally be expressed selectively (or even better specifically) in the cell or tissue of interest. Of the miRNAs investigated to date as potential biomarkers and therapeutic targets, however, almost all are evolutionarily conserved and expressed widely in most tissues and cell types. This observation is not peculiar to MPM, however, and can be seen by the predominance of relatively few miRNAs in functional preclinical studies of miRNA targeting approaches across cancer in general. Following the discovery of miRNAs in mammalian cells, the majority of human miRNAs identified were highly conserved, and this is reflected by most entries in the mirbase database. However, in a series of recent papers, a large number of cell- and tissue specific miRNAs were identified from RNA-seq data via computational approaches (83–85). These new miRNAs exhibit similar GC content and genome distribution to conserved miRNAs, and the expression of a number has been validated via RT-qPCR. While few have been characterized on a functional level, they nonetheless represent a rich source of potential biomarkers and therapeutic targets. Most recently, a study compared specific miRNA expression in lung cancer and MPM, demonstrating highly specific expression of a number of miRNAs that may prove able to assist with differential diagnosis (86). As a number are either highly expressed in MPM or present at lower levels, they represent candidate tumor suppressors and oncomiRs. Ongoing research will be required to determine whether they are altered in MPM carcinogenesis and to elucidate their functions.

### Alternative Delivery Approaches

Increasing evidence supports the concept that miRNA mimics represent a valid approach to therapy in MPM, but to date the only clinical experience remains the MesomiR-1 trial of TargomiRs (42). While the FDA approval of patisiran, and ongoing development of other siRNA- and miRNA-based drugs using liposomal or direct conjugation to targeting moieties underlines the potential for miRNAs to serve as cancer drugs (47), delivery to tumor cells *in vivo* remains a major hurdle (87). As the lipid-based delivery vehicles commonly used for double-stranded RNA drugs frequently accumulate in the liver, most siRNA- or miRNA-based drugs in development target hepatocytes. However, even with this selective delivery advantage, the miR-34a-based drug MRX34 targeting hepatocellular carcinoma or liver metastases was terminated due to unexpected immune-related adverse events (88). It is notable that seed sequence-mediated hepatotoxicity has been used to screen siRNA drug candidates prior to clinical development (89), but as the miR-34a mimic used did not cause immune events or hepatocyte damage in mouse models of liver cancer (90) and there are no published results describing these adverse events in more detail, the underlying cause remains unknown. Nevertheless, reaction to the liposomal vehicle, immune stimulation by double-stranded RNA or necrotic cell death may have played a role (88). The latter may be related to the toxicity of the GC-rich miR-34a seed, shown to preferentially downregulate survival genes and cause cell death in cancer cells (91).

In terms of vehicles for systemic delivery of miRNA mimics to organs other than the liver, few have reached an advanced stage of development. Most lipid- or nanoparticle-based systems are hampered by the inefficient escape from the endosomal system following endocytosis, meaning only a small fraction of the mimic molecules entering the cell are active in the cytoplasm (87). This is illustrated by studies with patisiran which suggest that of the 60% of the total dose that is delivered to the hepatocytes, only 3% is associated with the RISC machinery (92). An alternative approach gaining traction involves the use of extracellular vesicles (EVs) such as exosomes or microvesicles for miRNA delivery. Cells release a variety of EVs that contain a range of cellular molecules including miRNAs (93). Their ability to transfer miRNAs and mRNAs to recipient cells and influence gene expression was demonstrated in early studies (94–96), and their role in intercellular communication in cancer is now widely accepted (97). The ability of EVs to deliver miRNAs has subsequently been exploited as a potential vehicle method for miRNA mimics and siRNAs. An early study purified exosomes from dendritic cells engineered to express a neuron-specific targeting moiety, which were then electroporated with GAPDH siRNA (98). These exosomes were able to cross the blood-brain barrier and reduce GAPDH expression in neurons and other brain cells. In a similar approach, exosomes from HEK293 cells engineered to produce an EGFR-specific peptide ligand, and transfected with let-7a mimic, delivered let-7a mimic to EGFR-expressing breast cancer xenografts and inhibited their growth (99).

A clinically advanced example of this approach is represented by the delivery of engineered exosomes loaded with mutant KRAS-targeting siRNAs to inhibit pancreatic cancer (100). Following intraperitoneal injection, exosomes loaded with siRNA accumulated to a greater extent in the pancreas, and were also more growth inhibitory in a KRAS mutant orthotopic tumor xenografts model compared with liposomes carrying the same siRNA. This was demonstrated to be a result of increased retention in the circulation and cellular uptake via micropinocytosis, due in part to endogenous transmembrane proteins (100). Although many questions remain surrounding the large-scale production and purification of EVs (92), a phase I trial of KRAS siRNA-loaded exosomes, dubbed iExosomes, is scheduled to start in early 2020 [NCT03608631].

In the context of the potential use of EVs to deliver miRNAs to MPM, three recent studies are of particular relevance. The first described showed that a miR-15a mimic loaded into isolated exosomes via electroporation was able to decrease its target BCL2 when delivered to human monocytes *in vitro*, and increased miR-15a in mouse alveolar macrophages *in vivo* (101). The second study investigated the distribution of exosome-delivered miR-126 in a co-culture system combining mesothelial or MPM cells with endothelial cells and fibroblasts. MiR-126 accumulated in endothelial cells co-cultured with mesothelial or miR-126 sensitive MPM cells, but it accumulated in fibroblasts when the system contained miR-126 insensitive MPM cells (102), suggesting a role for miR-126 in controlling angiogenesis. Finally, the third study described methotrexate (MTX) delivery using autologous tumor cell-derived membrane microparticles (TMPs) in a malignant pleural effusion (MPE) model, and was based on the homotypic adhesion properties of cancer cell TMPs that increase their uptake by cancer cells (103). Immunocompetent mice with MPE resulting from intrapleural inoculation of tumor cells, were treated with MTX-TMPs via intrapleural administration. These mice developed fewer foci, had reduced MPE volume and survived longer than those treated with empty TMPs or MTX alone (103). These results were extended in a pilot clinical trial of 11 lung cancer patients with malignant pleural effusions. Treatment with MTX-TMPs proved safe, most patients had reduced MPE volume and symptomatic improvements, and assessment of fluid revealed fewer tumor cells. The continuing phase I trial aims to recruit 90 patients and has an expected completion date of December 2019 [NCT02657460]. As exosomes (and presumably other EVs) are numerous in MPE from MPM patients (104) and in cell-conditioned medium secreted from MPM cells (105, 106), and these are preferentially taken up by the cell of origin, they represent a potential vehicle for therapeutic miRNA delivery.

## Conclusions

Multiple lines of evidence support the continued development of miRNA-based therapies for MPM. Numerous miRNAs have been demonstrated to contribute to cancer hallmarks in MPM cells *in vitro*, and manipulating their expression using miRNA mimics or inhibitors can inhibit the proliferation and invasion of MPM cells and their interaction with stromal and immune cells. In addition to targeted systemic delivery with minicells, local delivery via intrapleural administration of miRNA mimics complexed with atelocollagen or encapsulated in (patient-derived) EVs have enormous potential for the treatment of MPM. Continued clinical investigation and optimization of methods for EV preparation, purification and miRNA loading will be needed to realize the potential of these novel treatment approaches.

## Author Contributions

GR wrote the first draft of the manuscript. GR and TJ generated the figures. GR, TJ, and NZ revised the manuscript and approved the submitted version.

### Conflict of Interest

The authors declare that the research was conducted in the absence of any commercial or financial relationships that could be construed as a potential conflict of interest. The reviewer LM declared a past co-authorship with one of the authors GR to the handling Editor.
